# SNPs near the cysteine proteinase cathepsin O gene *(CTSO)* determine tamoxifen sensitivity in ERα-positive breast cancer through regulation of BRCA1

**DOI:** 10.1371/journal.pgen.1007031

**Published:** 2017-10-02

**Authors:** Junmei Cairns, James N. Ingle, Lawrence D. Wickerham, Richard Weinshilboum, Mohan Liu, Liewei Wang

**Affiliations:** 1 Department of Molecular Pharmacology and Experimental Therapeutics, Mayo Clinic, Rochester, Minnesota, United States of America; 2 Division of Medical Oncology, Mayo Clinic, Rochester, Minnesota, United States of America; 3 Section of Cancer Genetics and Prevention, Allegheny General Hospital, Pittsburgh, Pennsylvania, United States of America; 4 National Surgical Adjuvant Breast and Bowel Project, Pittsburgh, Pennsylvania, United States of America; St. Jude Children's Research Hospital, UNITED STATES

## Abstract

Tamoxifen is one of the most commonly employed endocrine therapies for patients with estrogen receptor α (ERα)-positive breast cancer. Unfortunately the clinical benefit is limited due to intrinsic and acquired drug resistance. We previously reported a genome-wide association study that identified common SNPs near the *CTSO* gene and in *ZNF423* associated with development of breast cancer during tamoxifen therapy in the NSABP P-1 and P-2 breast cancer prevention trials. Here, we have investigated their roles in ERα-positive breast cancer growth and tamoxifen response, focusing on the mechanism of CTSO. We performed *in vitro* studies including luciferase assays, cell proliferation, and mass spectrometry-based assays using ERα-positive breast cancer cells and a panel of genomic data-rich lymphoblastoid cell lines. We report that CTSO reduces the protein levels of BRCA1 and ZNF423 through cysteine proteinase-mediated degradation. We also have identified a series of transcription factors of BRCA1 that are regulated by CTSO at the protein level. Importantly, the variant CTSO SNP genotypes are associated with increased CTSO and decreased BRCA1 protein levels that confer resistance to tamoxifen. Characterization of the effect of both *CTSO* SNPs and *ZNF423* SNPs on tamoxifen response revealed that cells with different combinations of *CTSO* and *ZNF423* genotypes respond differently to Tamoxifen, PARP inhibitors or the combination of the two drugs due to SNP dependent differential regulation of BRCA1 levels. Therefore, these genotypes might be biomarkers for selection of individual drug to achieve the best efficacy.

## Author summary

Many studies have demonstrated that germline genetic variation can contribute to both breast cancer disease risk and treatment response. However, the underlying mechanisms associated with these biomarkers often remains understudied. As part of functional genomic studies following up a case-control genome-wide association study (GWAS) performed with the large and influential National Surgical Adjuvant Breast and Bowel Project P-1 and P-2 SERM breast cancer prevention trials, we investigated the top GWAS SNPs in CTSO gene on chromosome 4 and mechanisms of CTSO involvement in the regulation of BRCA1 and response to therapy. We showed that, based on individual’s genotype, CTSO contributes differentially to tamoxifen response in ERα-positive (ER+) breast cancer cells by regulating ZNF423 and BRCA1levels and that PARP inhibitors can effectively restore tamoxifen sensitivity in subjects with unfavorable genotypes of CTSO and ZNF423 associated with tamoxifen resistance. Our work highlights the potential value of a new biomarker signature involving CTSO and ZNF423-related SNPs for selection of tamoxifen or PARP inhibitors.

## Introduction

Approximately 80% of breast tumors express estrogen receptor α (ER) [1–3], a receptor that binds and mediates many of the effects of estrogens. Estrogen signaling is known to modulate several processes relevant to breast cancer cell proliferation, predominately as a result of the activity of ER as a transcription factor [4]. Therefore, selective estrogen receptor modulators (SERMs) such as tamoxifen have been widely used clinically in endocrine therapies for patients with ERα-positive (ERα+) breast cancer [5–7]. Tamoxifen is not only effective in the treatment of ERα+ breast cancer, but it is also effective in the chemoprevention of breast cancer [8, 9]. However, resistance to tamoxifen therapy also occurs in that 22.7% of patients treated in the adjuvant setting had recurrence of breast cancer by 10 years in a meta-analysis, and in the prevention setting [10] tamoxifen reduces risk by 49%, but the number needed to treat to prevent one case of breast cancer is in excess of 50 [8]. Several mechanisms have been associated with resistance to tamoxifen [11, 12]. Of particular importance are the effects of estrogen/ER on BRCA1. The BRCA1 protein directly interacts with ERα and inhibits ERα transactivation and downstream signaling [13]. Decreased BRCA1 expression has been shown to be present in 30–40% of sporadic breast cancers [14]. BRCA1 deficiency is known to play a role in breast cancer development. Furthermore, decreased BRCA1 expression results in tamoxifen resistance by altering ERα co-regulator association in breast cancer cells [15]. These findings suggest that BRCA1 may regulate the response of ERα to its canonical ligand E2 and to tamoxifen, a compound known to exert either agonistic or antagonistic activity toward ERα in different cellular and tissue contexts [16]. In addition, BRCA1 is also known to play a major role in the DNA double-strand break (DSB) repair during the S and G2 phases by mediating homologous recombination (HR) to maintain replication fidelity and genome integrity [17]. Studies have demonstrated that BRCA1 dysfunction results in the lack of HR and markedly sensitizes cells to the inhibition of PARP enzymatic activity, which seemed to be attributable to the persistence of DNA lesions that are normally repaired by homologous recombination [18, 19]. Therefore, genetic factors that might contribute to BRCA1 regulation could significantly affect response to drugs like SERMs and PARP inhibitors.

Our previous case-control genome-wide association study (GWAS) performed with samples from the NSABP P-1 and P-2 breast cancer SERM chemoprevention trials identified two SNP signals that were associated with breast cancer risk, including one in which the variant SNP genotype near the *CTSO* gene was associated with increased risk for the development of breast cancer and a second signal for which the variant SNP genotype in the *ZNF423* gene was associated with decreased risk for the development of breast cancer in women treated with tamoxifen or raloxifene [20]. ZNF423 appeared to be a transcription factor that regulated BRCA1 expression in an estrogen-dependent fashion, while CTSO also showed weak estrogen-dependent induction of BRCA1 mRNA expression in a *CTSO* SNP-dependent fashion [20]. In a separate study, it was also shown that the variant GG genotype for the *CTSO* rs10030044 SNP was an independent factor indicating a poor prognosis in ER+ breast cancer patients receiving adjuvant tamoxifen therapy [21], which suggested the involvement of this genetic locus in tamoxifen response. *CTSO*, cathepsin O, is a member of the cysteine protease family that is involved in cellular protein degradation and turnover. Another member, cathepsin D, has been associated with poor prognosis for breast cancer as a result of stimulation of breast cancer cell proliferation, fibroblast outgrowth, angiogenesis, breast tumor growth and metastasis formation [22]. Even though in our previous study, we have observed a correlation between CTSO and BRCA1 in an estrogen and SNP dependent fashion, how CTSO regulates BRCA1 remains unclear. In the current study, based on our prior findings [20], we investigated the possible role of CTSO in drug response and breast cancer risk as a result of the regulation of ZNF423 and BRCA1. Finally, we also explored the role of both *ZNF423* and *CTSO* SNP genotypes to help selection of tamoxifen and PARP inhibitors.

## Results

### CTSO SNPs associated with breast cancer risk

Our previous GWAS involved 592 cases and 1171 matched controls selected from the 33,000 participants enrolled in the NSABP P-1 and P-2 breast cancer prevention trials identified two SNPs on chromosome 4 (rs10030044 and rs4256192) that were associated with breast cancer risk, with odds ratios of 1.42 and 1.44 respectively [20]. To gain a comprehensive understanding of the contribution of genetic variants in that region, together with the two top genotyped SNPs, based on our previous imputation results [20], we chose additional six imputed SNPs associated with increased risk for the development of breast cancer (OR 1.42–1.45) with adjusted p-values < 5.00E-6 (rs6835859, rs4550865, rs10030044, rs62328155, rs11737651, rs6810983, rs4256192, rs11724342). All eight SNPs were located at 5′ of the *CTSO* gene. These variant SNP genotypes are common with MAFs ranging from 0.39 to 0.45. We then performed linkage disequilibrium (LD) analysis and the analysis showed that all 8 SNPs were in significant linkage with each other. The top two genotyped SNPs, rs10030044 and rs4256192 were in strong LD (r^2^ = 0.78). The SNP rs10030044 was also in strong LD with the three imputed SNPs: rs6835859 (r2 = 1), rs4550865 (r^2^ = 1), and rs6810983 (r^2^ = 1), while the rs4256192 SNP was in strong LD with the other three imputed SNPs: rs11724342 (r2 = 1), rs62328155 (r2 = 1) and rs11737651 (r^2^ = 1). Because of the importance of understanding breast cancer risk and because P-1 and P-2 are the largest breast cancer chemoprevention trials ever performed, we pursued the possible functional implications of these SNP signals.

### Expression Quantitative Trait Loci (eQTL) analysis

**Fig 1:**
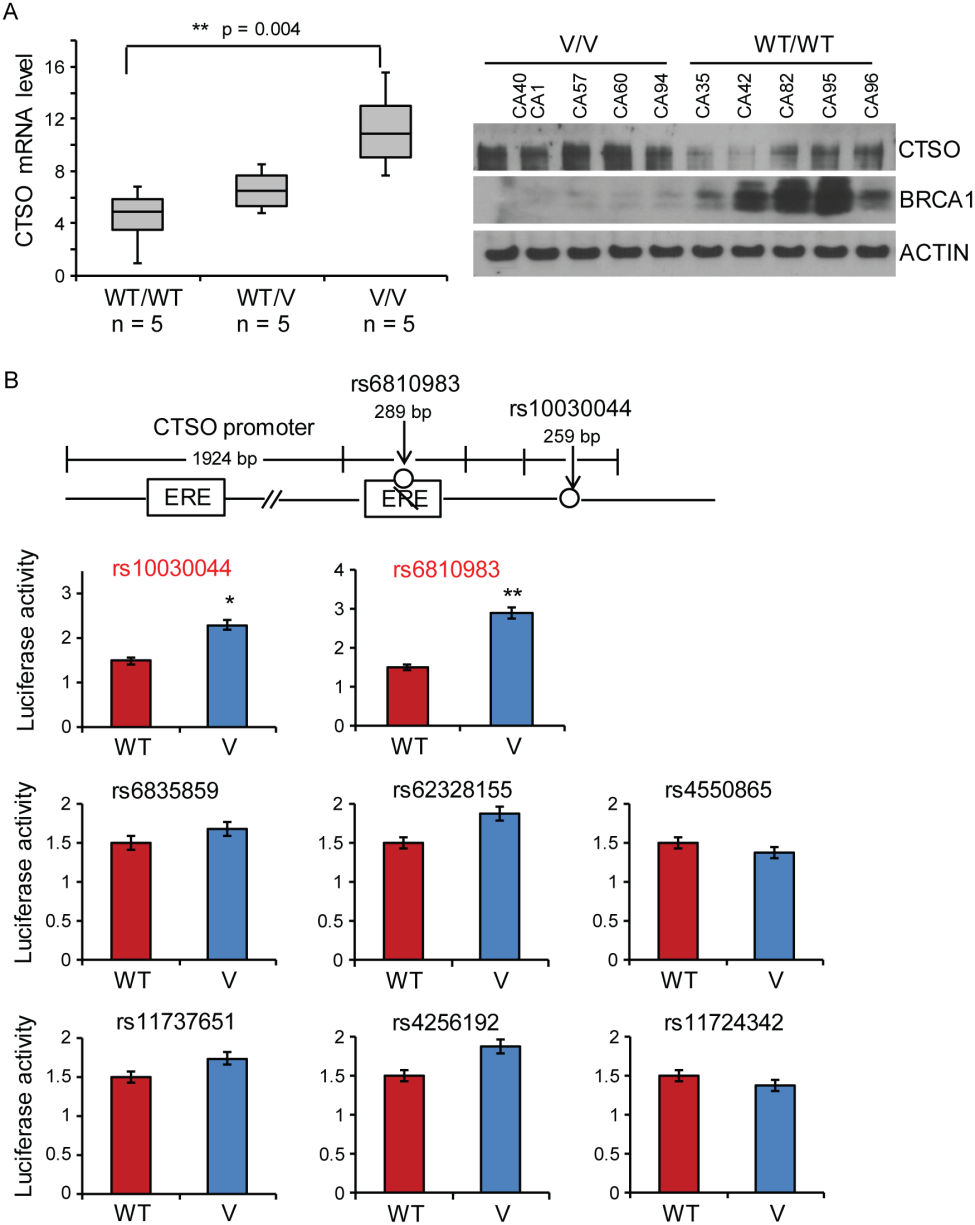
*CTSO* SNPs function. (A) *CTSO* expression is increased for cells of tissues homozygous for variant (V/V) or with heterozygous (WT/V) SNP genotypes as compared to homozygous wild type (WT/WT) in LCLs. (B) Luciferase assay results comparing the top 8 WT and variant SNP genotypes effects on transcriptional activities indicate increased transcription for the variant rs10030044 and rs6810983 SNPs in ZR75-1 cells. * = p < 0.05; ** = p < 0.01.

We began by analyzing the top 8 SNPs for their associations with expression levels of all genes including CTSO within 1 Mb up- and downstream of the SNPs of interest using the Genotype-Tissue Expression (GTEx) database. Although, we did not find eQTL relationships between these SNPs and CTSO in normal breast tissue in GTEx, significant eQTL associations between the SNPs and CTSO were present in stomach, skin, pancreas, and testis. The variate SNP was associated with higher CTSO expression (p = 0.0077–4.3E-7). We did not observe eQTL relationships between these SNPs and CTSO at baseline in our panel of LCLs for which we had genome-wide genotype data and mRNA expression data [23]. Because 94.2% of the participants on P-1 and P-2 were Caucasian, our GWAS was restricted to only Caucasian subjects [20]. Therefore, we randomly selected LCLs from Caucasians that were either homozygous wild type (WT) or variant for the SNPs 5’ of *CTSO* to validate the eQTL relationships in a setting mimicking the estrogenic environment in patients. These LCLs were grown in medium containing charcoal-treated serum to deplete the levels of endogenous steroids and supplemented with physiological concentrations of E2. CTSO mRNA and protein were higher in LCLs homozygous for the variant genotype as compared with LCLs homozygous for the WT genotype (p<0.05; Fig 1A). However, the induction of CTSO mRNA was more significant in the WT than variant cells, consistent with our previous finding [20], even though the variant cells had higher baseline level of CTSO (S1 Fig).

### CTSO SNPs and CTSO transcription

We next determined which of the SNPs 5’ of *CTSO* might influence expression. Our previous study suggested that the expression of *CTSO* was estrogen-dependent, and only the rs6810983 SNP disrupted an estrogen response element (ERE) for the variant SNP genotype [20]. We decided to directly determine the possible role of these eight SNPs in transcription regulation using luciferase reporter gene assays performed in ZR75-1 breast cancer cells. Specifically, we cloned a 200 bp DNA sequence that included either WT or variant sequence for each of the eight SNPs, together with the *CTSO* promoter, into the pGL3 basic reporter plasmid. We then transfected these constructs into the ER+ cell line, ZR75-1 cells in a normal medium with 10% FBS. Cells transfected with constructs with variant genotypes for rs10030044 and rs6810983 SNPs displayed 2–3 fold greater luciferase activity than did those transfected with constructs with WT SNP sequences, indicating increased transcriptional activity (Fig 1B)—compatible with the results in LCLs.

### CTSO mediates BRCA1 degradation via a proteolytic mechanism

**Fig 2:**
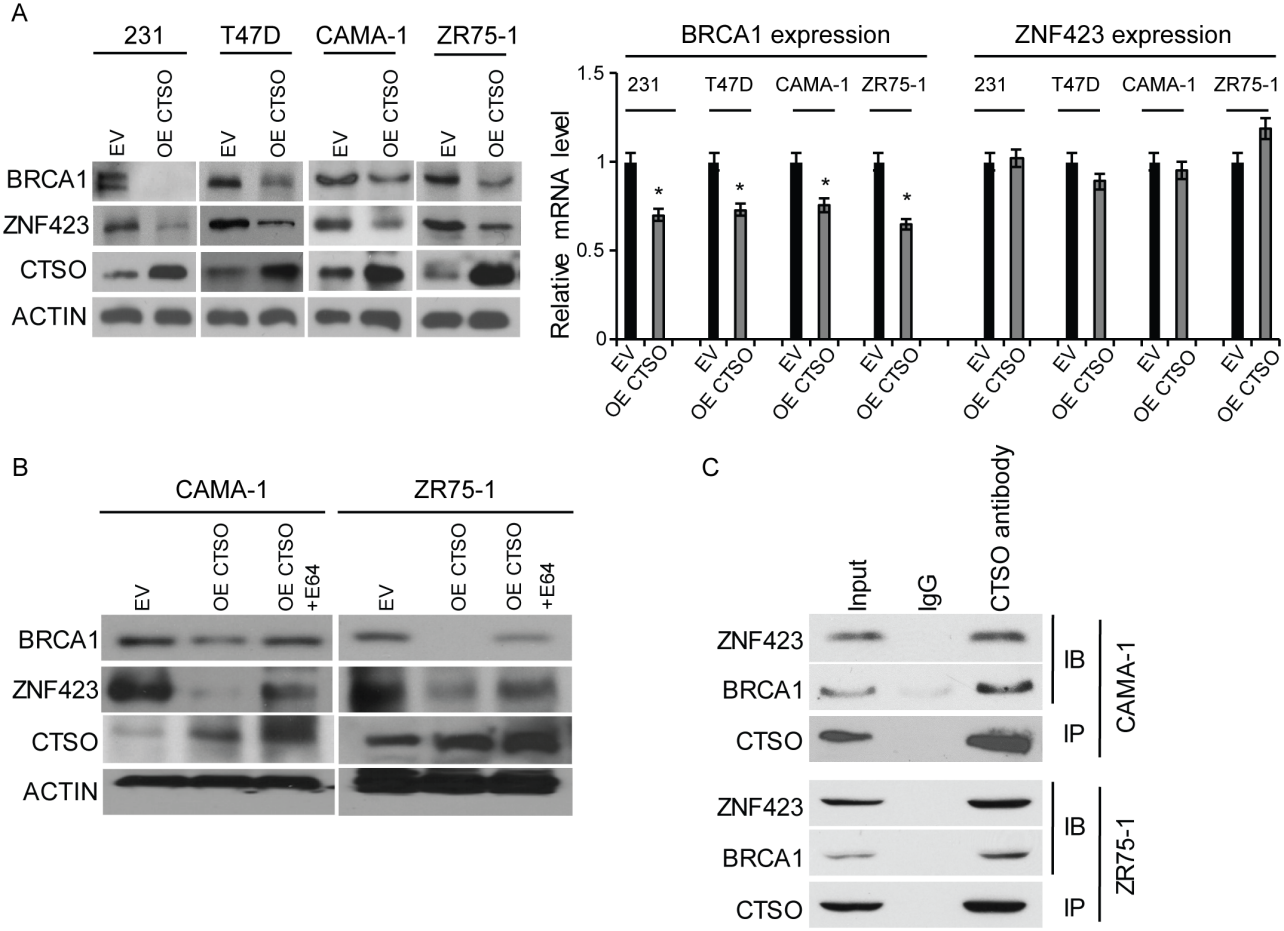
CTSO is responsible for the degradation of BRCA1 and ZNF423. (A) Over-expression of CTSO down-regulated BRCA1 and ZNF423 proteins in multiple human breast cancer cell lines. Over-expression of CTSO decreased BRCA1, but not ZNF423 mRNA expression levels. Error bars represent SEM. * = p < 0.05. (B) CTSO regulated BRCA1 and ZNF423 stability in a cysteine protease-dependent manner. (C) Direct endogenous interaction between CTSO and BRCA1 as well as ZNF423 by immunoprecipitation.

We then determined the possible functional effect of CTSO on BRCA1 based on our previous finding [20]. We genotyped the *ZNF423* SNP and *CTSO* SNP in a panel of breast cancer cell lines and chose T47D, CAMA-1, and ZR75-1 cell lines carrying homozygous genotypes for *ZNF423* and *CTSO* SNPs (S1 Table) for further functional study. When CTSO was overexpressed significantly in T47D, CAMA-1, and ZR75-1 cells, there was a striking decrease of BRCA1 protein levels as well as protein levels for the BRCA1 transcription factor, ZNF423, in all the cell lines tested (Fig 2A, left panel). To determine how generalizable this phenomenon might be, we also measured the level of BRCA1 protein in triple negative MDA-MB-231 breast cancer cells. In agreement with ER+ breast cancer cell line data, BRCA1 protein was significantly decreased after overexpressing CTSO in triple negative breast cancer cells (Fig 2A, left panel). Quantitative RT-PCR revealed excellent transfection efficiency of CTSO in all of the cell lines, with modest but statistically significant decreases in BRCA1 transcript levels (Fig 2A, right panel), while ZNF423 mRNA remained unchanged after CTSO overexpression (Fig 2A, right panel). Next, we asked whether CTSO might influence BRCA1 and ZNF423 protein stability through its cysteine proteases activity. Overexpression of CTSO decreased ZNF423 and BRCA1 protein levels in CAMA-1 and ZR75-1 cells, while treatment with the cathepsin inhibitor E-64 resulted in increased levels of BRCA1 and ZNF423 protein (Fig 2B). Previous work has largely focused on *CTSO* SNP-dependent estrogen induction of CTSO and BRCA1 mRNA in LCLs. Consistent with our previous finding [20], both CTSO and BRCA1 mRNA was moderately induced by E2 in LCLs with WT CTSO SNP genotype (S1 Fig). However, in this study, we further demonstrated that, more importantly, CTSO can also directly regulate BRCA1 protein turnover in breast cancer cells.

Since CTSO is able to stimulate BRCA1 and ZNF423 protein degradation, we determined the possible interaction between CTSO and BRCA1or ZNF423. Immunoprecipitation using CTSO antibody showed endogenous interaction of CTSO with BRCA1 and ZNF423 (Fig 2C). These results indicated that CTSO regulates BRCA1 and ZNF423 protein stability through a cysteine protease- mediated degradation pathway—at least in part.

### CTSO affects BRCA1 expression through the degradation of BRCA1 transcription regulators

**Fig 3:**
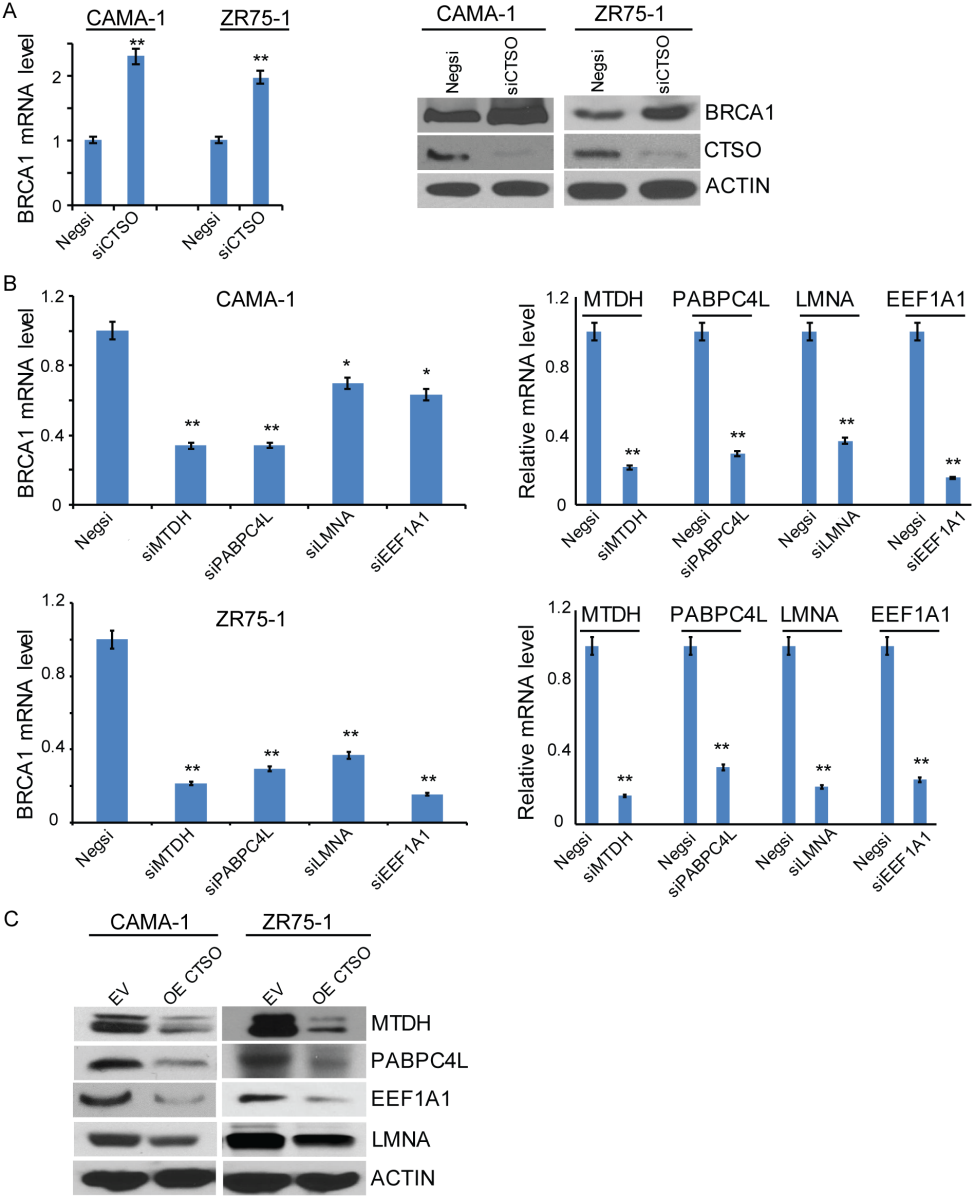
CTSO is responsible for the degradation of BRCA1 transcription regulators. (A) Knock down of CTSO up-regulated BRCA1 mRNA and protein level in CAMA1 and ZR75-1 cell lines. (B) Knock down of *MTDH*, *PABPC4L*, *LMNA*, and *EEF1A1* down-regulated BRCA1 mRNA level. The knock down efficiency was determined by qRT-PCR. * = p < 0.05; ** = p < 0.01. (C) Over-expression of CTSO down-regulated MTDH, PABPC4L, LMNA, and EEF1A1 protein levels in CAMA1 and ZR75-1 cells.

We next examined possible mechanisms by which CTSO might influence BRCA1 transcription. We first confirmed that knockdown of CTSO resulted in increased BRCA1 expression, both at the mRNA and protein levels in both CAMA-1 and ZR75-1 cells (Fig 3A). Our previous GWAS study had reported that ZNF423 binds to the 5′-flanking region of BRCA1 and regulates BRCA1 transcription [20]. We also showed in the present study that CTSO interacts with ZNF423, leading to ZNF423 degradation (Fig 2), suggesting that CTSO may regulate BRCA1 transcription partially through its effect on ZNF423. In order to identify additional factors involved in the CTSO-dependent regulation of BRCA1 transcription, we performed mass spectrometry screening of a pool of proteins that co-precipitated with CTSO. During this process, we identified 130 proteins that interacted with CTSO (S2 Table). We then interrogated the Cancer Genome Atlas (TCGA) breast cancer data [24] for possible relationships between the expression of BRCA1 and these 130 genes, and identified 20 genes that were associated with BRCA1 with p< 1E-05 (S3 Table). We then knocked down these 20 genes to determine the effect on BRCA1 levels (S2 Fig), and found that knockdown of 4 out of the 20 genes, *MTDH*, *PABPC4L*, *LMNA*, and *EEF1A1*, resulted in striking decreases of BRCA1 mRNA expression level (Fig 3B), consistent with the TCGA data that showed positive correlations between these 4 genes and BRCA1. Furthermore, in CAMA-1 and ZR75-1 cells, overexpression of CTSO decreased expression of all four genes (Fig 3C), which could explain the down-regulation of BRCA1 mRNA level when overexpressing CTSO (Fig 2A). In summary, these results indicate that the up-regulation of CTSO could reduce BRCA1 levels by promoting the cysteine protease—mediated degradation of MTDH, PABPC4L, LMNA, and EEF1A1 protein levels in addition to the effect on ZNF423 that we had already identified, all of which regulate BRCA1 transcription. Thus, it appears that tumor expression of CTSO may play a role in the regulation of BRCA1 transcription in addition to having an effect on BRCA1 protein degradation.

### Effect of CTSO-mediated degradation of BRCA1 on growth arrest and tamoxifen response

**Fig 4:**
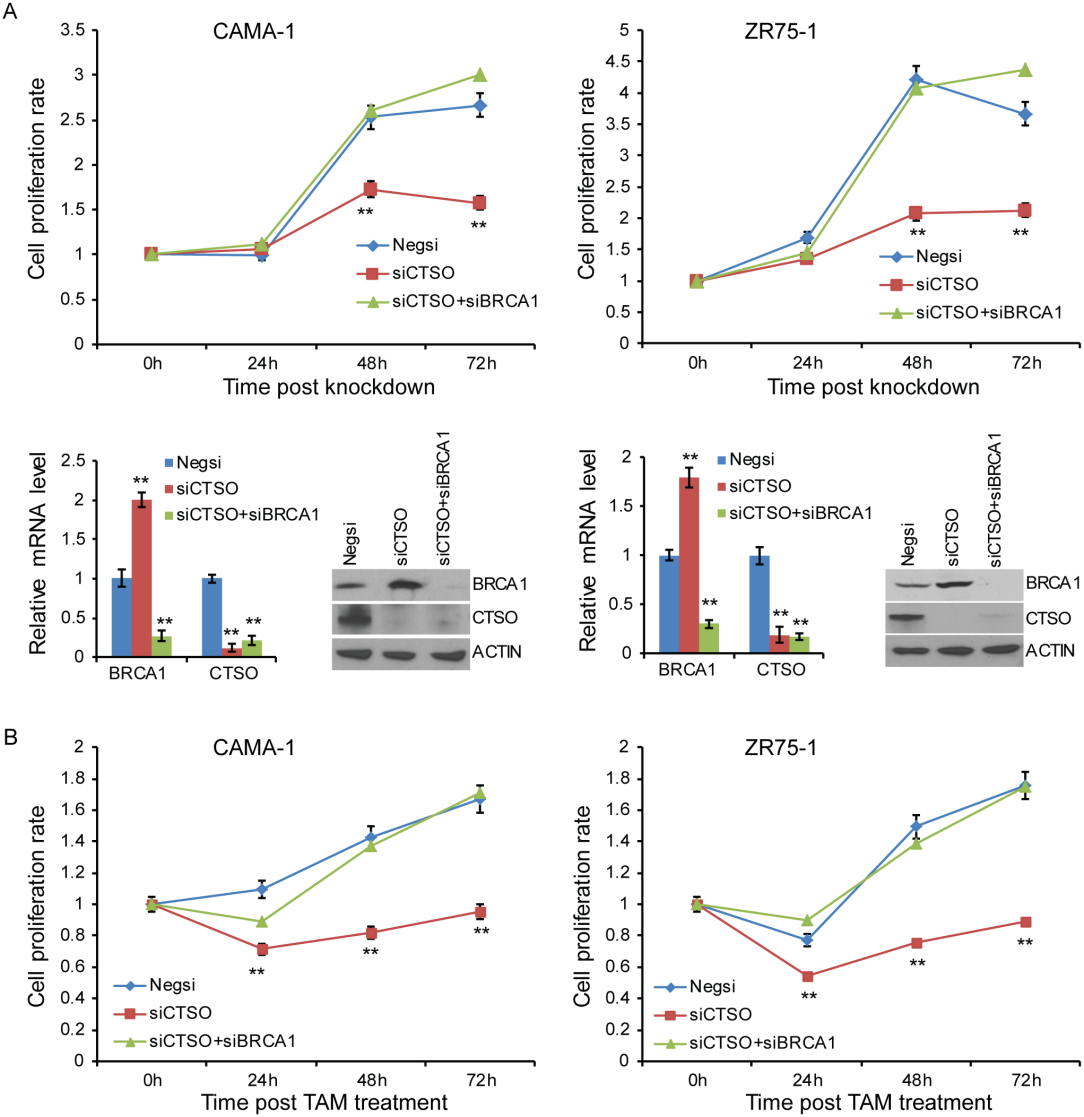
CTSO regulates ER+ breast cancer cell proliferation and tamoxifen response. (A) Knock down of CTSO decreased proliferation in CAMA1 and ZR75-1 cell lines through BRCA1. Error bars represent SEM; ** P< 0.01 compared to baseline (negative control). The knock down efficiency was determined by qRT-PCR and western blot. (B) Knock down of CTSO conferred sensitivity to tamoxifen through BRCA1. Knock down of BRCA1 abrogated CTSO effects on proliferation in CAMA1 and ZR75-1 cells in the presence of 4-OH TAM. Error bars represent SEM. * = p < 0.05; ** = p < 0.01.

We hypothesized that, because CTSO regulates BRCA1 stability, it may play a role in endocrine resistance. Previous studies demonstrated that BRCA1over-expression can inhibit cell proliferation by activating p21^WAF1/CIP1^ [25, 26]. We had demonstrated that CTSO regulates the stability of BRCA1 (Fig 2). Therefore, we next determined whether the down-regulation of CTSO inhibited cell proliferation in breast cancer cells due to the up-regulation of BRCA1. BRCA1 protein increased after CTSO knockdown in CAMA-1 and ZR75-1 cells (Fig 4A, lower panel). Depletion of CTSO inhibited cell growth compared with negative siRNA transfected control cells (Fig 4A, upper panel). To further confirm that the CTSO effect on cell proliferation was mediated through the regulation of BRCA1, we knocked down BRCA1 in cells with down-regulation of CTSO. Knockdown of BRCA1 in CTSO-depleted cells resulted in the abrogation of decreased proliferation due to CTSO depletion in both cell lines (Fig 4A, upper panel). We next tested the effect of CTSO on tamoxifen treatment based on the observations from our previous study [20] and others. In the presence of 100 nM 4OH-tamoxifen (4OH-TAM), CTSO-deficient cells exhibited increased sensitivity to 4OH-TAM compared with negative siRNA-transfected control cells (Fig 4B), and BRCA1 might be responsible for the increased sensitivity since BRCA1 depletion in siCTSO cells significantly decreased 4OH-TAM sensitivity (Fig 4B). These results demonstrated that depletion or inhibition of CTSO can increase BRCA1 levels with potential therapeutic effects, resulting in growth arrest.

### ZNF423 and CTSO SNP genotypes and tamoxifen response

Since our previous study had identified *ZNF423* and *CTSO* SNPs that were associated with breast cancer risk [20], both of which appeared to regulate BRCA1, we examined their joint effect on cell proliferation in the presence of tamoxifen or E2 treatment. We utilized a model system consisting of 300 individual human LCLs (100 European-American, 100 African-American and 100 Han Chinese-American subjects). The “Human Variation Panel” that had been SNP genotyped previously and has repeatedly demonstrated its value as a platform to study genetic variants [20, 27, 28]. Specifically, we selected 4 groups of LCLs to perform 4OH-TAM treatment:

Group1: 4 LCLs with homozygous wild type (WT) genotypes for both *CTSO* and *ZNF423* SNPs—*CTSO* W/*ZNF423* W;
Group2: 4 LCLs with homozygous variant genotypes for both *CTSO* and *ZNF423* SNPs—*CTSO* V/*ZNF423* V;
Group3: 4 LCLs with homozygous variant genotypes for *CTSO* SNPs and homozygous WT for *ZNF423* SNPs—*CTSO* V/*ZNF423* W;
Group4: 4 LCLs with homozygous WT genotypes for *CTSO* SNPs and homozygous variant for *ZNF423* SNPs—*CTSO* W/*ZNF423* V.

**Fig 5:**
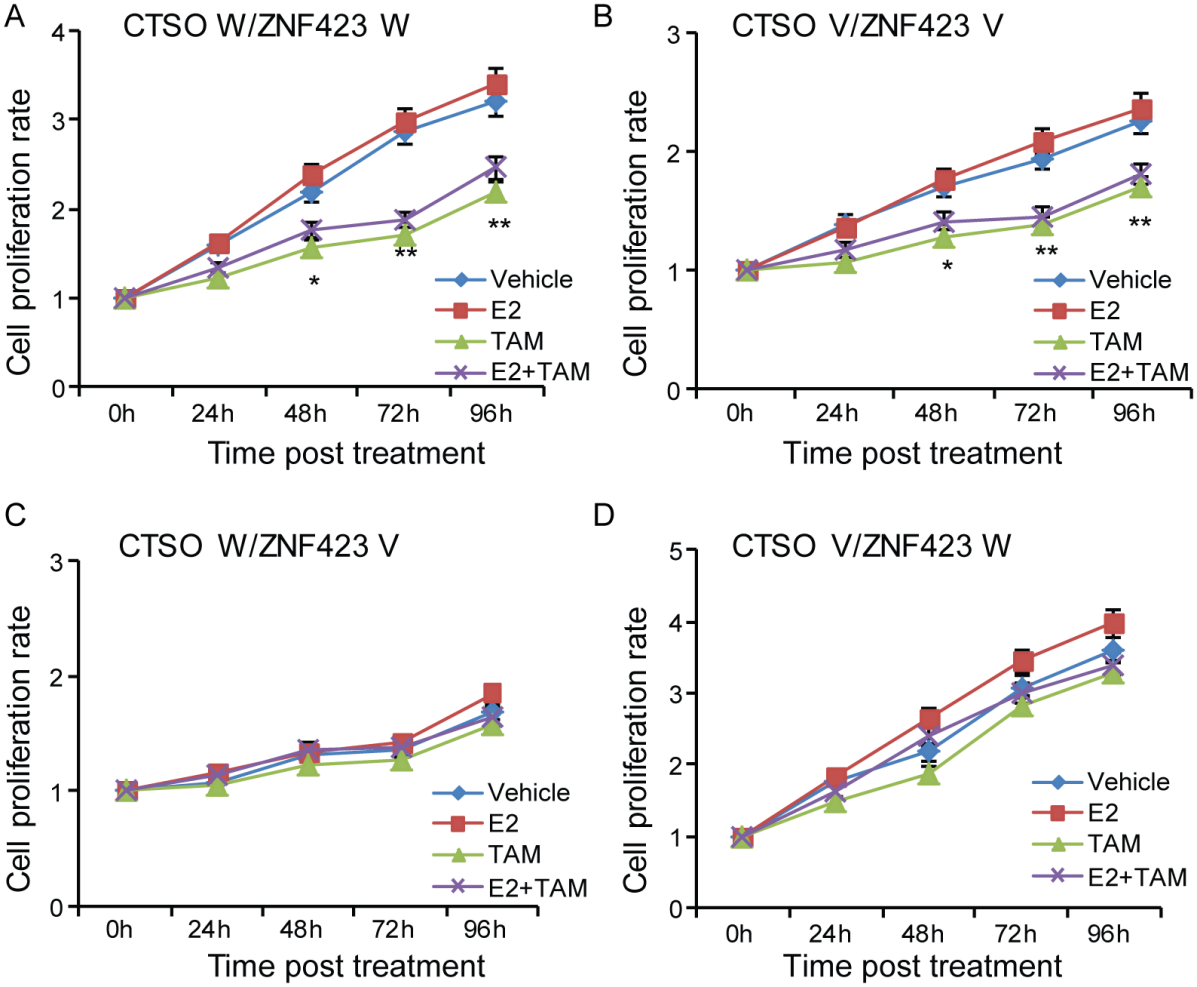
*ZNF423* and *CTSO* SNPs joint effects on tamoxifen response. LCLs carrying *CTSO* W/*ZNF423* W (A) and *CTSO* V/*ZNF423* V (B) respond to 4-OH TAM treatment comparing to vehicle. LCLs with *CTSO* W/*ZNF423* V (C) or *CTSO* V/*ZNF423* W (D) failed to respond to 4-OH TAM treatment comparing to vehicle. Cells were treated with vehicle, E2, TAM, or E2 plus TAM as described in material and methods. Error bars represent SEM. n = 4 for each group. * = p < 0.05; ** = p < 0.01.

**Fig 6:**
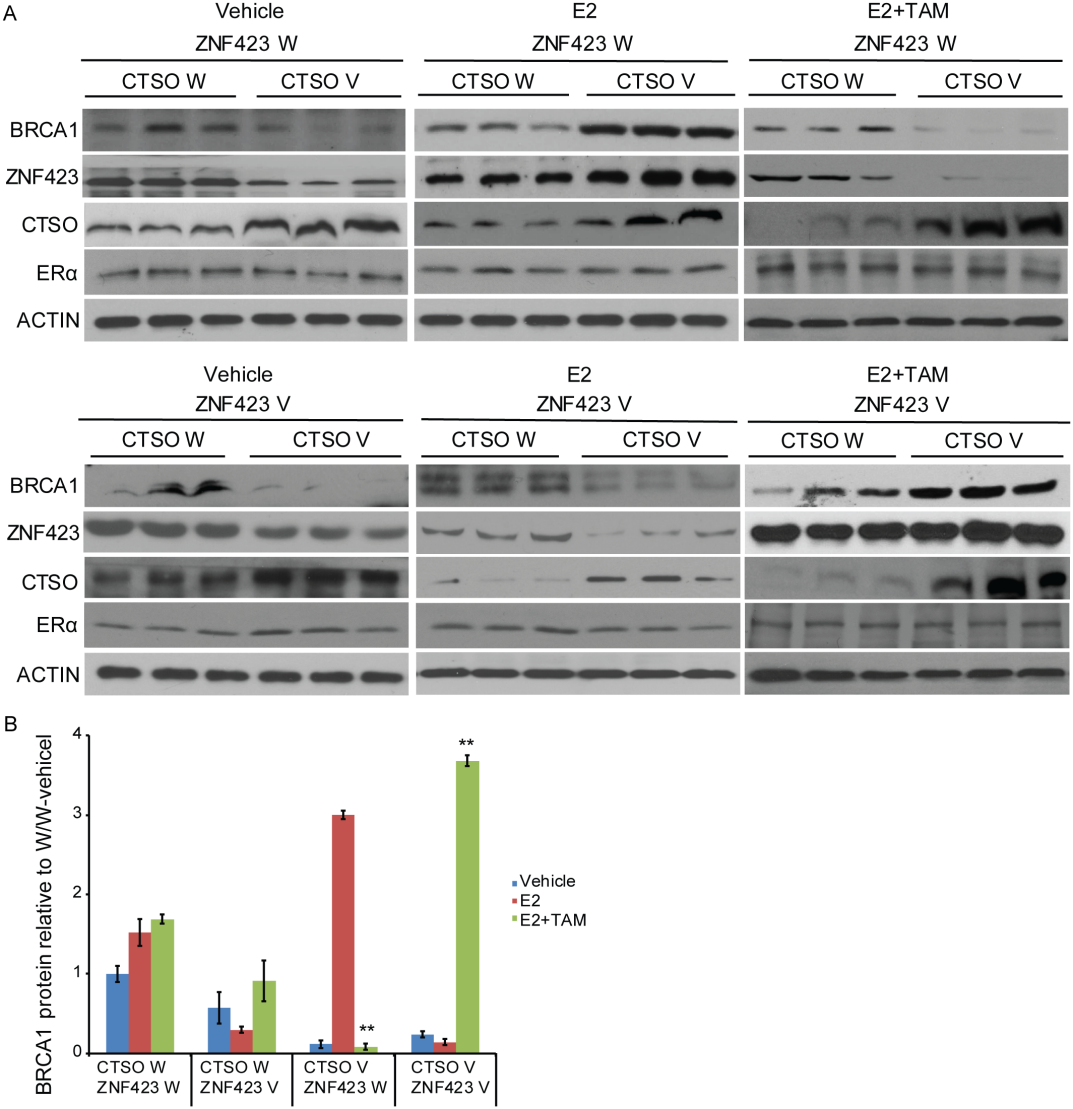
*ZNF423* and *CTSO* SNPs joint effects on BRCA1 protein level during tamoxifen therapy. (A) 4 groups of LCLs carrying *CTSO* W/*ZNF423* W, *CTSO* V/*ZNF423* W, *CTSO* W/*ZNF423* V, and *CTSO* V/*ZNF423* V were treated with vehicle, E2, or E2 plus TAM as described in material and methods. BRCA1 protein was analyzed by western blotting. (B) Quantitative analysis of BRCA1 protein level of the results from (A) using Image J. BRCA1 protein levels were first normalized to ACTIN for each genotype and treatment, and then the level of BRCA1 relative to *CTSO* W/*ZNF423* W vehicle treated group was shown for all the genotype combination and treatments. Error bars represent ± SEM of three independent experiments. ** = p < 0.01.

Notably, in the presence of 4OH-TAM, the growth of *CTSO* WT/*ZNF423* WT and *CTSO* V/*ZNF423* V cells decreased significantly (Fig 5A and 5B, and Table 1) suggesting that the therapeutic effects of tamoxifen are seen mainly in the *CTSO* WT/*ZNF423* WT and *CTSO* V/*ZNF423* V groups, not the *CTSO* WT/*ZNF423* V and *CTSO* V/*ZNF423* WT groups (Fig 5C and 5D, and Table 1). We also measured BRCA1, CTSO and ZNF423 protein levels in cells with different *ZNF423* SNP and *CTSO* SNP combinations (Fig 6). The estradiol-, 4OH-TAM -dependent and SNP-dependent regulation of *BRCA1* protein level was more pronounced against the background of homozygous variant for the *CTSO* SNP. BRCA1 protein level in the *CTSO* V / *ZNF423* WT group was significantly upregulated in the presence of E2 and then decreased upon addition of 4OH-TAM treatment (Fig 6). The opposite effects on BRCA1 protein level upon treatment of E2 or E2 plus 4OH-TAM were observed in *CTSO* V / *ZNF423* V group compared with *CTSO* V / *ZNF423* WT group (Fig 6B). The higher BRCA1 level in *CTSO* V / *ZNF423* V group compared to the *CTSO* V / *ZNF423* WT group in the presence of 4OH-TAM could explain the tamoxifen response seen in *CTSO* V / *ZNF423* V group, but not in *CTSO* V / *ZNF423* WT group (Figs 4 and 5B and 5D). In the presence of TAM, cells with *CTSO* W */ ZNF423* W genotype were also showed relatively higher BRCA1 levels, even though with this genetic background the baseline BRCA1 was higher compared with other genotype groups (Fig 6B). Therefore, cells with *CTSO* W / *ZNF423* W also benefit from TAM treatment (Fig 5A). We also measured ER level in these four groups of LCLs upon different treatment to account for its potential impact, and did not observe difference in ER level among the four genotype combination groups, furthermore, E2 and TAM treatment did not change the level of ER compared to vehicle treatment for each genotype combination (Fig 6). Therefore, the ZNF423 and CTSO SNPs-dependent effects on TAM response were not due to ER expression level.

**Table 1:** ZNF423 and CTSO SNP joint analysis on 4OH-TAM response with or without olaparib.

Genotype		CTSO	
		WT (AF = 55%)	V (AF = 45%)
	WT (AF = 61%)	PGC = 55%*55%*61%*61% = 11.26% Tam responsive, No PARPi benefit	PGC = 45%*45%*61%*61% = 7.54% **Tam non-responsive**, **Responsive with PARPi added**
ZNF423			
	V (AF = 39%)	PGC = 55%*55%*39%*39% = 4.60% Tam non-responsive, No PARPi benefit	PGC = 45%*45%*39%*39% = 3.08% Tam responsive, No PARPi benefit

Prevalence of the genotype combination (PGC) is calculated based on homozygous allele frequency (AF) for ZNF423 SNP (Minor allele frequency = 39%) and homozygous AF for CTSO SNP (Minor allele frequency = 45%).

When compared the cell proliferation in the presence of different treatments among different genotypes, cells with *CTSO* V/*ZNF423* W showed the fastest growth rate, regardless of whether they received no treatment, estradiol (E2) alone, 4OH-TAM alone, or the combination of E2 plus 4OH-TAM (S3 Fig), while cells with *CTSO* WT/*ZNF423* V grew slowest among all genotype combination groups in all treatment groups (S3 Fig). This was consistent with our previous finding that the odds ratios for *CTSO* V/*ZNF423* W (OR = 5.71) was the highest, and that for *CTSO* WT/*ZNF423* V (OR = 1.00) was the lowest for breast cancer risk in the P-1, P-2 trials [20].

### ZNF423 and CTSO SNP genotypes and breast cancer proliferation with PARP inhibitor therapy

**Fig 7:**
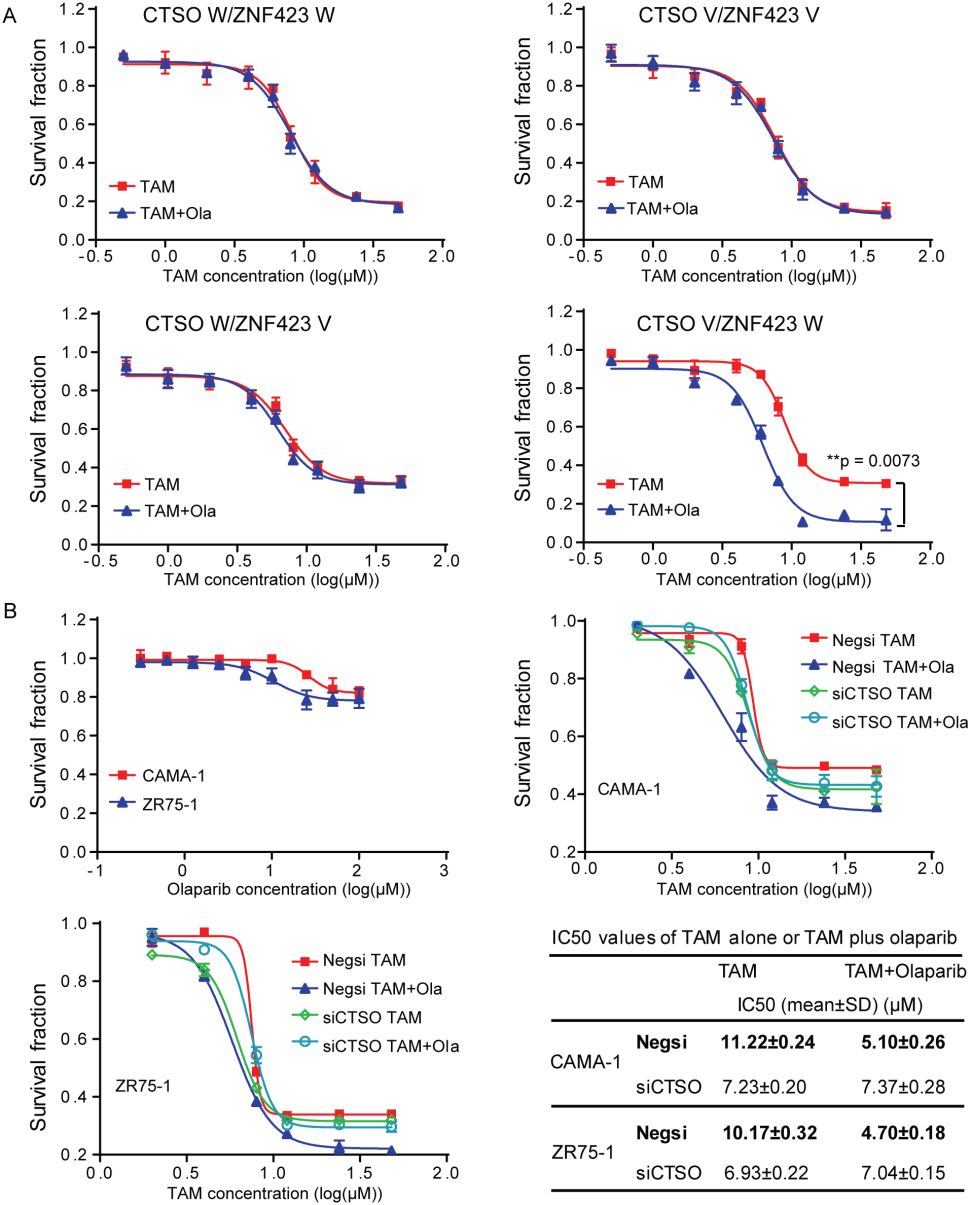
PARP inhibitor enhances tamoxifen sensitivity that allows bypass of CTSO SNP effect on BRCA1. (A) Olaparib (Ola) sensitizes cells with high CTSO level to 4-OH TAM treatment. Olaparib does not change 4-OH TAM sensitivity in the 4-OH TAM-sensitive LCLs, *CTSO* W/*ZNF423* W and *CTSO* V/*ZNF423* V comparing to 4-OH TAM alone. Olaparib sensitizes 4-OH TAM-resistant LCLs with *CTSO* V/*ZNF423* W to 4-OH TAM treatment comparing to 4-OH TAM alone. (B) CAMA-1 and ZR75-1 cells are resistant to olaparib alone treatment. Olaparib sensitizes ER+ breast cancer cells with high CTSO level (negative siRNA transfected) to 4-OH TAM treatment comparing to cells with lower CTSO (siCTSO transfected). Error bars represent SEM. * = p < 0.05; ** = p < 0.01. IC50 values for 4-OH TAM were calculated using GraphPad Prism 7 software (n = 3, mean ± SEM).

Loss of BRCA1 function leads to defects in the HR DNA repair pathway, which renders cells more sensitive to PARP inhibitors [29–32]. In BRCA1/2 mutated cells, the DSBs at the replication fork caused by PARP inhibitor treatment cannot be repaired, resulting in synthetic lethality and cell death. We have shown that the LCL *CTSO* WT/*ZNF423* WT (Fig 5A) and *CTSO* V/*ZNF423* V (Fig 5B) groups respond to 4OH-TAM treatment but not the *CTSO* WT/*ZNF423* V (Fig 5C) and *CTSO* V/*ZNF423* WT (Fig 5D) groups (Table 1). In addition, comparing the two 4OH-TAM-resistant groups, *CTSO* WT/*ZNF423* V cells showed higher BRCA1 level upon 4OH-TAM treatment than *CTSO* V/*ZNF423* WT cells (Fig 6B). As a result, we hypothesized that the combination of a PARP inhibitor and 4OH-TAM might achieve better therapeutic outcomes in the *CTSO* V/*ZNF423* WT group that displayed lower levels of BRCA1. To determine the effect of a PARP inhibitor in this setting, we treated 4OH-TAM-responsive *CTSO* WT/*ZNF423* WT and *CTSO* V/*ZNF423* V LCLs as well as 4OH-TAM-resistant *CTSO* WT/*ZNF423* V and *CTSO* V/*ZNF423* WT LCLs with either 4OH-TAM alone or 4OH-TAM plus the PARP inhibitor, olaparib. Olaparib did not increase 4OH-TAM sensitivity in the two 4OH-TAM-responsive *CTSO* WT/*ZNF423* WT and *CTSO* V/*ZNF423* V groups (Fig 7A, upper panel, and Table 1). However, olaparib significantly sensitized the 4OH-TAM-resistant *CTSO* V/*ZNF423* WT cells to tamoxifen treatment, but not the *CTSO* WT/*ZNF423* V cells (Fig 7A, lower panel, and Table 1). The differential effects of olaparib in the two 4OH-TAM-resistant groups can be explained, at least partially, by the differences in BRCA1 levels (Fig 6B). Upon 4OH-TAM treatment, the 4OH-TAM-resistant *CTSO* V/*ZNF423* WT cells had lower BRCA1 levels compared with the *CTSO* WT/*ZNF423* V cells, resulting in sensitization by combining olaparib with 4OH-TAM. The 4OH-TAM-resistant *CTSO* WT/*ZNF423* V cells had high level of BRCA1, consistent with olaparib having little effect.

We also confirmed the therapeutic effect of the combination of olaparib and 4OH-TAM in ER+ breast cancer cells, CAMA-1 and ZR75-1 that had WT BRCA1 and were resistant to olaparib (Fig 7B). Knock down of CTSO resulted in striking increases of BRCA1 protein level (Fig 3A), therefore, the addition of olaparib did not increase 4OH-TAM sensitivity (Fig 7B). However, olaparib significantly increased 4OH-TAM sensitivity in cells transfected with negative control siRNA due to lower baseline BRCA1 level comparing with CTSO knockdown cells (Fig 7B, p<0.05). 4OH-TAM showed the 50% inhibitory concentration (IC50) of 11.22 μM for CAMA-1, and 10.17 μM for ZR75-1 cells transfected with negative control siRNA respectively. The IC50 of 4OH-TAM decreased significantly when co-treated with olaparib in negative control siRNA transfected CAMA-1 and ZR75-1 cells (CAMA-1: IC50 = 5.10±0.26μM; ZR75-1: IC50 = 4.70±0.18 μM) (Fig 7B, p<0.05). In summary, these results indicated that the down-regulation of CTSO could increase BRCA1 levels, resulting in decreased cell growth and potential therapeutic effects.

## Discussion

Understanding intrinsic or acquired resistance to endocrine therapy in the treatment or prevention of breast cancer is of great importance [11]. Tamoxifen is still widely used to treat ER+ breast cancer and, along with the SERM raloxifene, are the only FDA-approved drugs for prevention of breast cancer in high-risk women. Our previous GWAS study identified SNPs on chromosome 4, near the *CTSO* gene that were associated with increased risk for the development of breast cancer during five years of breast cancer prevention therapy with tamoxifen or raloxifene in the NSABP P-1 and P-2 breast cancer prevention trials [20]. Recently, Hato *et al* reported a correlation between the variant (GG) genotype for *CTSO* rs10030044 and shorter disease-free survival, and shorter overall survival in hormone receptor-positive breast cancer patients receiving adjuvant tamoxifen therapy [21]. Multivariate Cox regression analysis revealed that this genotype was an independent factor indicating a poor prognosis in hormone receptor-positive breast cancer patients receiving adjuvant tamoxifen therapy [21]. Our previous work has largely focused on *CTSO* SNP-dependent estrogen induction of CTSO and BRCA1 mRNA in LCLs [20]. However, the exact mechanism by which CTSO regulates BRCA1 is not clear. Adding E2 can have multiple effects on both CTSO and BRCA1. Additionally, the different combinations of ZNF423 and CTSO genotypes also add additional complexity of regulation of downstream proteins like BRCA1. Therefore, in this study, we focused on the possible mechanisms of *CTSO* gene involvement in the regulation of BRCA1 and response to therapy in different genotype background. The data presented here demonstrated a possible role for *CTSO* in resistance to tamoxifen, since the down-regulation of CTSO led to the inhibition of cell growth and increased BRCA1 protein level through both regulation of BRCA1 transcription factors and BRCA1 protein degradation in ER+ breast cancer cells. In addition, we obtained evidence that the addition of PARP inhibitor to tamoxifen could reverse resistance to tamoxifen in breast cancer cells with higher levels of *CTSO* gene expression. Genotypes for *ZNF423* and *CTSO* could regulate gene expression in an estrogen or tamoxifen-dependent fashion, in turn, influencing downstream BRCA1 levels. Therefore, based on individual genotypes, we could potentially select different treatments to achieve the best outcomes, i.e. precision breast cancer prevention or therapy.

CTSO is a cysteine protease. This class of proteases mediates catabolism of intracellular proteins and selectively activates extracellular protein degradation, macrophage function, and bone resorption [33]. Cysteine proteases have been shown to function extracellularly as well as intracellularly [34, 35], and have been suggested as potential targets for anti-cancer therapy [35, 36]. Cathepsins B, D, H, L, or L2 are thought to play a role in several cancers [37–39]. The role of cathepsins in resistance to cancer therapy is an area of emerging interest [40, 41]. Our current studies demonstrate the mechanisms underlying CTSO-mediated tamoxifen resistance in ER+ breast cancer.

Specifically, our functional genomic studies demonstrated that, among the top 8 SNPs near the *CTSO* gene from our previous GWAS, the rs10030044 and rs6810983 SNPs could regulate *CTSO* gene expression, and these SNPs were associated with higher *CTSO* gene expression levels (Fig 1). We next examined the possible relationship between CTSO expression and that of BRCA1, a gene known to be induced by estrogen exposure through a mechanism that has remained unclear [42, 43].

We found a negative correlation between CTSO and BRCA1 protein levels (Fig 2). Based on our observations of the effect of CTSO on both BRCA1 protein and mRNA levels, we first hypothesized that CTSO might regulate BRCA1 through a cysteine protease -mediated pathway, which we experimentally confirmed by treatment with a cysteine protease inhibitor (Fig 2B). Furthermore, regulation of the transcription of BRCA1 by CTSO was found to be through the regulation of *ZNF423* [20], *MTDH* [44, 45], *PABPC4L* [46], *LMNA* [47], and *EEF1A1* [48] transcription factors (Figs 2 and 3). MTDH (AEG-1) regulates c-MYC through PLZF, and c-MYC induces BRCA1 gene expression [44, 45]. PABPC4L (Poly A Binding Protein Cytoplasmic 4 Like) is a member of PABP family. PABP recognizes the 3′ mRNA poly (A) tail and plays critical roles in eukaryotic translation initiation and mRNA stabilization/degradation [46, 49]. LMNA (A-type lamin) has been shown to control transcription of BRCA1 [47]. EEF1A1 (translation elongation factor 1-alpha 1) affects gene expression through regulating mRNA stability [48], and could also regulate BRCA1 through E2F1 [50, 51]. Therefore, the ultimate BRCA1 protein level is regulated by CTSO at both transcription as well as protein levels. Decreased BRCA1 has been shown to abolish tamoxifen suppression of cell proliferation [15]. We showed that down-regulation of CTSO increased BRCA1 protein level and inhibited proliferation of ER+ cells with or without tamoxifen treatment (Figs 3 and 4). Inhibition could be restored by co-silencing BRCA1 and CTSO gene expression (Fig 4), suggesting that CTSO may regulate cell proliferation and tamoxifen response through BRCA1.

Our initial GWAS had identified SNPs associated with decreased (*ZNF423*) and increased (*CTSO*) risk for breast cancer occurrence [20], both of which appeared to regulate BRCA1. The joint odds ratios for the development of breast cancer while on SERM therapy for five years for these two sets of SNPs ranged from 1.00 for women homozygous for both sets of favorable, low-risk alleles, to 5.71 for women homozygous for unfavorable, high- risk alleles for both *ZNF423* and *CTSO*. In the present study, we also evaluated their joint effect on cell proliferation in the presence of tamoxifen in LCLs carrying different combinations of *ZNF423* and *CTSO* genotypes. We found that the cells homozygous for the favorable alleles of both *CTSO* and *ZNF423* (*CTSO* W/*ZNF423* V) proliferated slowest, while cells homozygous for the unfavorable alleles of both *CTSO* and *ZNF423* (*CTSO* V/*ZNF423* W) proliferated fastest at baseline without treatment (S3 Fig). With tamoxifen treatment, these two genotype groups remained the slowest-growing (favorable) and fastest-growing (unfavorable) groups among the four different genotype groups (S3 Fig), suggesting that tamoxifen had no further effect on the proliferation of cells with these two genotype groups (Fig 5C and 5D). At the mechanistic level, tamoxifen benefit is partially determined by the induction of BRCA1 level. Cells homozygous for one favorable allele and the other unfavorable allele (*CTSO* W/*ZNF423* W, and *CTSO* V/*ZNF423* V groups) responded to tamoxifen treatment (Fig 5A and 5B, Table 1), both of which showed high induction of BRCA1 levels in the presence of TAM (Fig 6B), indicating that patients with these two genotype groups might benefit the most from tamoxifen treatment. For the two tamoxifen-nonresponsive cell groups, in cells carrying *CTSO* V/*ZNF423* W genotypes, PARP inhibitor treatment restored tamoxifen sensitivity (Fig 7A). However, a PARP inhibitor did not sensitize tamoxifen in the other tamoxifen- non responsive cells with the *CTSO* W/*ZNF423* V genotypes, which might be due to the higher level of BRCA1 level in those cells (Figs 6 and 7A, Table 1). Consistent with a previous study [52], we found that cells with lower BRCA1 level due to higher CTSO were very sensitive to PARP inhibition (Fig 7B). The combination of genotyping for *CTSO* SNPs and *ZNF423* SNPs offers the potential for the stratification of ER+ breast cancer patients into different drug response subgroups. Specifically, the use of PARP inhibitors in combination with tamoxifen in patients carrying the *CTSO* V/*ZNF423* W SNP genotypes offers an opportunity for improving tamoxifen sensitivity and prognosis in these patients. The findings of no efficacy for tamoxifen alone or in combination with a PARP inhibitor in patients with the favorable SNP genotype profile of *CTSO* W/*ZNF423* V raises the possibility that alternative approaches to prevention in low-risk patients should be studied in such patients.

### Conclusions

In conclusion, we present evidence in the present study that CTSO is a new factor of importance for tamoxifen efficacy as a chemopreventive agent in women at high risk of developing breast cancer as well as evidence for a potential mechanism by which this effect involves BRCA1. The underlying mechanisms identified require validation and further refinement but they also provide pharmacogenomic insights into tamoxifen as a preventative agent. We have demonstrated that a PARP inhibitor, which can effectively restore tamoxifen sensitivity in tamoxifen—resistant ER+ breast cancer cells, might be a potentially promising addition to tamoxifen as a combination regimen for patients carrying the *CTSO* V*/ZNF423* W SNP genotype. As a result, our study has revealed a new potential biomarker signature involving *CTSO* and *ZNF423*-related SNPs for the therapeutic stratification of patients at high risk for the development of breast cancer.

## Materials and methods

### Chemicals and reagents

Dulbecco's minimum essential medium (DMEM), glutamine and penicillin/streptomycin/glutamine stock mix were purchased from Life Technologies, Inc. (Carlsbad, CA, USA). Fetal bovine serum (FBS) and charcoal-stripped FBS were from Invitrogen (Carlsbad, CA, USA). L-trans-Epoxysuccinyl-leucylamido (4-guanidino) butane (E-64) was from Sigma-Aldrich (St. Louis, MO USA). CTSO, MTDH, PABPC4L, LMNA, EEFiA1and control small interfering RNAs (siRNA) were purchased from Dharmacon (Thermo Scientific Dharmacon, Inc.). CTSO plasmid was purchased from OriGene (Rockville, MD, USA). Affinity purified rabbit and mouse antibodies against human BRCA1 and CTSO were from Santa Cruz Biotechnologies (Santa Cruz, CA, USA). ZNF423 antibody was purchased from Abcam (Cambridge, MA, USA). Actin, MTDH, PABPC4L, LMNA, and EEFiA1 antibodies were from cell signaling (Danvers, MA, USA). For standard PCR, HotStart Taq Plus DNA Polymerase was used (Qiagen, Germantown, MD, USA). Reagents and primers for real time PCR were purchased from Qiagen (Valencia, CA, USA). The protease inhibitor cocktail kit was obtained from Pierce Biotechnology (Rockford, IL, USA). 17β-estradiol (E2) and 4-hydroxytamoxifen (OH-TAM) were purchased from Sigma Aldrich (Saint Louis, MO USA). Olaparib was from Selleckchem (Houston, TX, USA).

### Cell lines

Lymphoblastoid cell lines (LCLs) with known genotypes for the chromosome (chr) 4 CTSO SNPs were cultured in RPMI 1640 media containing 15% (vol/vol) FBS (Invitrogen, San Diego, CA). T47D, ZR75-1, CAMA-1, MDA-MB-231 cell lines were obtained from American Type Culture Collection (ATCC) (Manassus, VA). T47D and ZR75-1 were cultured in RPMI-1640 (Grand Island, NY) containing 10% fetal bovine serum (FBS). CAMA-1 cells were cultured in Eagle's Minimum Essential Medium containing FBS to a final concentration of 10%. MDA-MB-231 cells were cultured in Leibovitz's L-15 Medium containing 10% FBS at 37°C without CO2.

### CTSO reporter gene assays

Luciferase reporter gene constructs containing various SNP genotypes were generated by PCR based mutagenesis. Specifically, a 1924 bp segment of the CTSO promoter containing ERE was PCR amplified with the primers: 5’- TAAGCAGATATCACTGACATCATGCCACACCT’ and 5- ACGATGCTGAGATTGACCCTAAGCTTTAAGCA -3’ and was cloned into the EcoRV and HindIII sites of pGL3 basic plasmid to make the pGL3-CTSO construct. A 150–250 bp DNA segment that included the rs10030044, rs6810983, rs6835859, rs4550865, rs62328155, rs11737651, and rs4256192 SNPs respectively was also PCR amplified using primers as described in S1 File.

These fragments were cloned into the KpnI and NheI sites upstream of the CTSO promoter sequence to make the plasmids pGL3-WT-CTSO or pGL3-V-CTSO. The WT SNP sequence was amplified with LCL genomic DNA as a template that was homozygous for this WT SNP genotype. This variant SNP sequence was amplified using LCL genomic DNA shown to be homozygous for the variant genotype as template. These 150 -250bp amplicons contained the rs10030044, rs6810983, rs6835859, rs4550865, rs62328155, rs11737651, and rs4256192 SNPs respectively.

T47D and ZR75-1 cells were then seeded in triplicate in 12-well cell culture plates at a concentration of 10^5^ cells / well. After 24 h, the cells were transfected using Lipofectamine 2000 (Invitrogen) with 4 μg of the pGL3-WT-CTSO or pGL-V-CTSO constructs and 2 μg pRL-CMV encoding a CMV-driven renilla luciferase vector (Promega), together with the carrier DNA (pGL3 basic). Luciferase assays were performed 48 h after transfection using a luciferase reporter assay system (Promega). The renilla luciferase activity was used to correct for the transfection efficiency.

### Human variation panel LCLs

The human variation panel model system consists of LCLs from 300 healthy subjects (100 European-Americans, 100 African-Americans, and 100 Han Chinese-Americans). This panel was generated by the Coriell Institute (Camden, New Jersey). We genotyped all 300 cell lines for genome-wide SNPs using Illumina 550K and 510S SNP BeadChips (Illumina), and the Coriell Institute obtained Affymetrix SNP array 6.0 (Affymetrix) data for the same cell lines. These combined SNP genotype data (~1.3 million genotyped SNPs) were used to impute a total of approximately 7 million SNPs per cell line. This LCL model system has been used repeatedly to generate and/or test pharmacogenomic hypotheses arising from clinical GWAS [3, 12, 17–19, 53]. The application of these cell lines made it possible to evaluate the function of *CTSO* and *ZNF423* SNP genotypes. To study the effect of the SNP on CTSO expression, LCLs were cultured in base media containing 5% charcoal-stripped FBS for 24 hours and were subsequently cultured in FBS-free base media containing 0.1 nM E2 for another 48 hours. Cell lysates were used to perform Western blot analysis, and total RNA was isolated for qRT-PCR.

### Drug treatment and cell growth assay

Breast cancer cells were cultured in specific base media, as described above, supplemented with 10% FBS. 5000 cells were seeded in triplicate in 96-well plates, and were cultured in base media containing 5% (vol/vol) charcoal-stripped FBS for 24 hours and were subsequently cultured in FBS-free base media for another 24 hours. Cells were then transfected with either control siRNA or siRNA targeting *CTSO*. Twenty-four hours after transfection the media was replaced with fresh FBS-free base media and the cells were treated with 0.1 nM E2 for 24 hours, and then treated with 100 nM 4-OH- tamoxifen. Cell growth was measured at different time points (0, 24, 48, and 72 hours) post tamoxifen treatment using the BrdU Cell Proliferation Assay kit (Cell Signaling, Danvers, MA) at intervals of 24 h following the manufacturer's instructions. The plates were measured in a Safire2 microplate reader (Tecan AG, Switzerland).

LCLs selected based on *ZNF423* and *CTSO* genotypes were cultured in RPMI 1640 media (Cellgro) supplemented with 15% FBS. Cells were cultured in RPMI 1640 media containing 5% (vol/vol) charcoal-stripped FBS for 24 hours and were subsequently seeded in triplicate in 96-well plates and cultured in FBS-free RPMI 1640 media for another 24 hours before treatment. Cells were treated with 0.1 nM E2, 50nM tamoxifen, or the combination of both 0.1 nM E2 and 50nM tamoxifen. Cell growth was measured at different time points (0, 24, 48, 72, and 96 hours) post treatment using the CYQUANT Direct Cell Proliferation Assay (#C35012, Invitrogen) following the manufacturer’s instructions at intervals of 24 h. The plates were measured in a Safire2 microplate reader (Tecan AG, Switzerland).

### Transfection and gene silencing

Cells were plated at 70% confluence in culture medium supplemented with 10% FBS, and were transfected with empty vector or CTSO plasmid (OriGene) using lipofectamine 2000 (Invitrogen, Carlsbad, CA) according to the vendor's protocol. Cells were collected for protein analysis 48 hours after transfection. In some experiments, 24 hours after transfection, cells were treated with 10 μM E-64, a cysteine proteases inhibitor, for additional 24 hours. Cells were then collected for protein analysis.

Specific siGENOME siRNA SMARTpool reagents against a given gene as well as a negative control, siGENOME Non-Targeting siRNA, were purchased from Dharmacon Inc. (Lafayette, CO, USA). Cells were transfected with control siRNA, and specific siRNAs (10nM) in 96-well plates or 12-well plates using lipofectamine RNAiMAX (Invitrogen, Carlsbad, CA) according to the vendor's protocol. For the purpose of cell growth assay, cells were plated in base medium supplemented with 5% charcoal stripped FBS for 24 hours, and then cultured in FBS-free RPMI 1640 media for another 24 hours before transfection. Different treatments were started 24 hours after transfection. For the purpose of testing gene expression level, cells were transfected with control siRNA and specific siRNAs (10nM) in 12-well plates using lipofectamine RNAiMAX for 48 hours.

### Western blot

Breast cancer cells were harvested by trypsinization, lysed in SDS buffer. Cell lysates were heated to 95°C for five minutes. Protein samples (10 to 20 μg) were resolved by electrophoresis on 10% sodium dodecyl sulfate polyacrylamide gel electrophoresis (SDS-PAGE) gels and electrophoretically transferred to PVDF membranes (Millipore Corporation, Bedford, MA, USA). The blots were probed with the appropriate primary antibody and the appropriate horseradish peroxidase conjugated secondary antibody. The protein bands detected with the Pierce enhanced chemiluminescence Western blotting substrate (Thermo Scientific, Rockford, IL, USA) and were visualized using Geldoc (Bio-Rad Laboratories).

LCLs selected based on ZNF423 and CTSO genotypes were cultured in RPMI 1640 media containing 5% (vol/vol) charcoal-stripped FBS for 24 hours and were subsequently seeded in 6-well plates and cultured in FBS-free RPMI 1640 media for another 24 hours before treatment. Cells were treated with 0.1 nM E2, 50nM tamoxifen, or combination of both 0.1 nM E2 and 50nM tamoxifen for 48 hours and lysed in RIPA buffer supplemented with protease and phosphatase inhibitors. Cell lysates were used to perform Western blot analysis. Quantification of the blots was analyzed using Image J.

### Immunoprecipitation and immunoblotting

Cells were lysed in NETN buffer (20 mM Tris-HCl, pH 8.0, 100 mM NaCl, 1 mM EDTA, 0.5% Nonidet P-40) supplemented with protease and phosphatase inhibitors. Lysates were clarified by centrifugation (13,000 r.p.m., 20 min, 4°C) and 500 μg–1mg proteins were used per immunoprecipitation. Proteins were captured with 2 μg CTSO antibody and protein G-sepharose Fast-Flow (Sigma). Immunoprecipitation with mouse serum was used as negative controls. The immuno-complexes were then washed with NETN buffer three times followed by separation on SDS-PAGE. Proteins were resolved by SDS–PAGE, transferred onto PVDF membranes and probed using the appropriate primary and secondary antibodies coupled to horse-radish peroxidase.

### RNA isolation and quantitative real time PCR (qRT-PCR)

Total RNA was isolated from cultured cells with the QIAGEN RNeasy kit (QIAGEN Inc., Valencia, CA, USA), followed by qRT-PCR performed with the one-step Brilliant SYBR Green qRT-PCR master mix kit (Stratagene, La Jolla, CA, USA). Specifically, primers purchased from QIAGEN were used to perform qRT-PCR with the Stratagene Mx3005P real-time PCR detection system (Stratagene). All experiments were performed in triplicate with GAPDH as an internal control. Reverse-transcribed Universal Human Reference RNA (Stratagene) was used to generate a standard curve. Control reactions lacked the RNA template. The 2^-δδcycle threshold^ method was used for statistical data analysis.

### Cytotoxicity assay

Drugs were dissolved in DMSO, and aliquots of stock solutions were frozen at −80°C. Cytotoxicity assays were performed in triplicate at each drug concentration. Specifically, 4000 breast cancer cells were seeded in 96-well plates and were cultured in base media containing 5% (vol/vol) charcoal-stripped FBS for 24 hours and were subsequently cultured in FBS-free base media for another 24 hours. Cells were then transfected with either control siRNA or siRNA targeting *CTSO*. Twenty-four hours after transfection the media was replaced with fresh FBS-free base media and the cells were treated with 10 μL of tamoxifen at final concentrations of 0, 0.5, 1, 2, 4, 6, 8, 12, 24, and 48 μM with or without 10 μM olaparib. After incubation for an additional 72 hours, cytotoxicity was determined by quantification of DNA content using CYQUANT assay (#C35012, Invitrogen) following the manufacturer’s instructions. 100μL of CyQUANT assay solution was added, and plates were incubated at 37°C for one hour, and then read in a Safire2 plate reader with filters appropriate for 480 nm excitation and 520 nm emission.

LCLs selected based on *ZNF423* and *CTSO* genotypes were cultured in RPMI 1640 media containing 5% charcoal-stripped FBS for 24 hours and 5x10^4^ cells were subsequently seeded in triplicate in 96-well plates and cultured in FBS-free RPMI 1640 media for another 24 hours before treatment. Cells were treated with 10 μL of tamoxifen at final concentrations of 0, 0.5, 1, 2, 4, 6, 8, 12, 24, and 48 μM with or without 5 μM olaparib. After incubation for an additional 72 hours, cytotoxicity was determined by quantification of DNA content using CYQUANT assay.

### CTSO interacting protein detected by Mass spectrometry

ZR75-1 cells were transfected with CTSO plasmid. After 72 hr, cells were lysed by NETN buffer. Cell lysates were incubated with control IgG or CTSO antibody at 4°C for 4 hr, and then incubated with protein G-sepharose Fast-Flow for 2 hr. After washing with NETN buffer three times, bound proteins were eluted, and size fractionated by 10% SDS-PAGE. Coomassie-stained gel slices covering the entire molecular weight range were processed for analysis by mass spectrometer following a standard protocol at the Harvard Medical School Taplin Mass Spectrometry Facility.

### Statistical analysis

All data were presented as mean ± SD of at least three independent experiments. Statistical analysis was performed using SPSS22.0 and Prism 5 (GraphPad Software Inc., San Diego, CA, USA). Single-factor analysis of the variance test was used for comparisons among multiple groups, and a t-test was used for comparisons between two groups; P <0.05 was considered statistically significant.

## Supporting information

S1 File This file provides detailed materials and methods for the additional figures. (DOCX)

S1 Table Genotype for *CTSO* and *ZNF423* SNPs in a panel of breast cancer cell lines. (XLSX)

S2 Table 130 proteins interact with CTSO based on mass spectrometry screening. (XLSX)

S3 Table Among the 130 proteins interacting with CTSO, 20 associated with BRCA1 with p< 1E-05 in TCGA breast cancer database. (XLSX)

S1 Fig mRNA levels of CTSO and BRCA1 in LCLs with homozygous WT and variant CTSO SNP after exposure to increasing concentrations of estradiol (E2). Error bars represent SEM.
(TIF)

S2 Fig The effects of CTSO interacting genes on BRCA1 gene expression in breast cancer cells. (TIF)

S3 Fig *ZNF423* and *CTSO* SNPs joint effects on cell proliferation. (TIF)
