# Supplementary material for: SNPs near the cysteine proteinase cathepsin O gene (CTSO) determine tamoxifen sensitivity in ERα-positive breast cancer through regulation of BRCA1
Source: PLoS Genet. 2017 Oct 2;13(10):e1007031. doi: 10.1371/journal.pgen.1007031 (PMC5638617; doi:10.1371/journal.pgen.1007031)
Supplement: S1 File — (DOCX) [file pgen.1007031.s001.docx]

# S1 File

## Transfection and gene silencing

CAMA-1 and ZR75-1 cells were plated at 70% confluence in 6-well plates in culture medium supplemented with 10% FBS, and were transfected with negative control siRNA or specific siGENOME siRNA SMARTpool® reagents against a given gene (Dharmacon, Lafayette, CO, USA). Cells were transfected with control siRNA, and specific siRNAs (10nM) using lipofectamine RNAiMAX (Invitrogen, Carlsbad, CA) according to the vendor's protocol. 48 hours later, total RNA was isolated from cultured cells with the QIAGEN RNeasy kit (QIAGEN Inc., Valencia, CA, USA), followed by qRT-PCR performed with the one-step Brilliant SYBR Green qRT-PCR master mix kit (Stratagene, La Jolla, CA, USA). Specifically, primers purchased from QIAGEN were used to perform qRT-PCR with the Stratagene Mx3005P™ real-time PCR detection system (Stratagene). All experiments were performed in triplicate with GAPDH as an internal control, and replicated three times. The 2-δδcycle threshold method was used for statistical data analysis.

## CTSO Reporter gene assays primers

rs10030044 (200bp): 5’- TAAGCAGGTACCTTTAAATAGGGATGCCGTTTG -3’ and 5’- ACCTGGGCTGGAGTACAATGGCTAGCTAAGCA -3’

rs6810983 (147bp): 5’- TAAGCAGGTACCTTTCATGAGGATGCTCTGGTC -3’ and 5’- TGCTGGGATTGTATCCTTTCGCTAGCTAAGCA -3’

rs6835859 (251bp): 5’- TAAGCAGGTACC GCCACAGCCCACAGTATGA -3’ and 5’- TGAAAGTGCTATTTTCTGTTGGA GCTAAGCA -3’

rs4550865 (186bp): 5’- TAAGCAGGTACCGGAAGGGAAGGGAACAAAAA -3’ and 5’- CTGATGGCTAGAATGGTCGTGCTAAGCA -3’

rs62328155 (199bp): 5’- TAAGCAGGTACC ACCCTAAAATGAGGCCTTGAA -3’ and 5’- CACAACTCTGGAGTCACCTTTGGCTAAGCA -3’

rs11737651 (195bp): 5’- TAAGCAGGTACCGTGGTCTTTGCAGGTATTGG -3’ and 5’- CAATTTGCTTCCATTGCCTAAGCTAAGCA -3’

rs4256192 (205bp): 5’- TAAGCAGGTACCGCACTGTGGGAGGTATGGTT -3’ and 5’- TTCTGATGCATTTCTGGAAAGAGCTAAGCA -3’

**Lymphoblastoid Cell Lines (LCLs) Culture**

LCLs with known genotypes for the chromosome (chr) 16 ZNF423 SNP or chr 4 CTSO SNP were cultured in RPMI media containing 15% (vol/vol) FBS. Prior to E2 treatment, 2×107 cells from each cell line were cultured for 24 h in RPMI 1640 media containing 5% (vol/vol) charcoal stripped FBS, followed by culture in the same medium without FBS for another 24 h. All cells were then cultured for 24 h in 6-well plates, with RPMI 1640 media that contained 0, 0.0001, 0.001, 0.01, and 0.1 nM E2. Total RNA was isolated from the cells with the RNeasy mini kit (Qiagen). 200 ng of total RNA was then used to perform qRT-PCR with CTSO and BRCA1 primers (Qiagen). CTSO and BRCA1 expression levels were normalized on the basis of ACTIN expression in each cell line.
