# Supplementary figures and images for: SNPs near the cysteine proteinase cathepsin O gene (CTSO) determine tamoxifen sensitivity in ERα-positive breast cancer through regulation of BRCA1
Source: PLoS Genet. 2017 Oct 2;13(10):e1007031. doi: 10.1371/journal.pgen.1007031 (PMC5638617; doi:10.1371/journal.pgen.1007031)

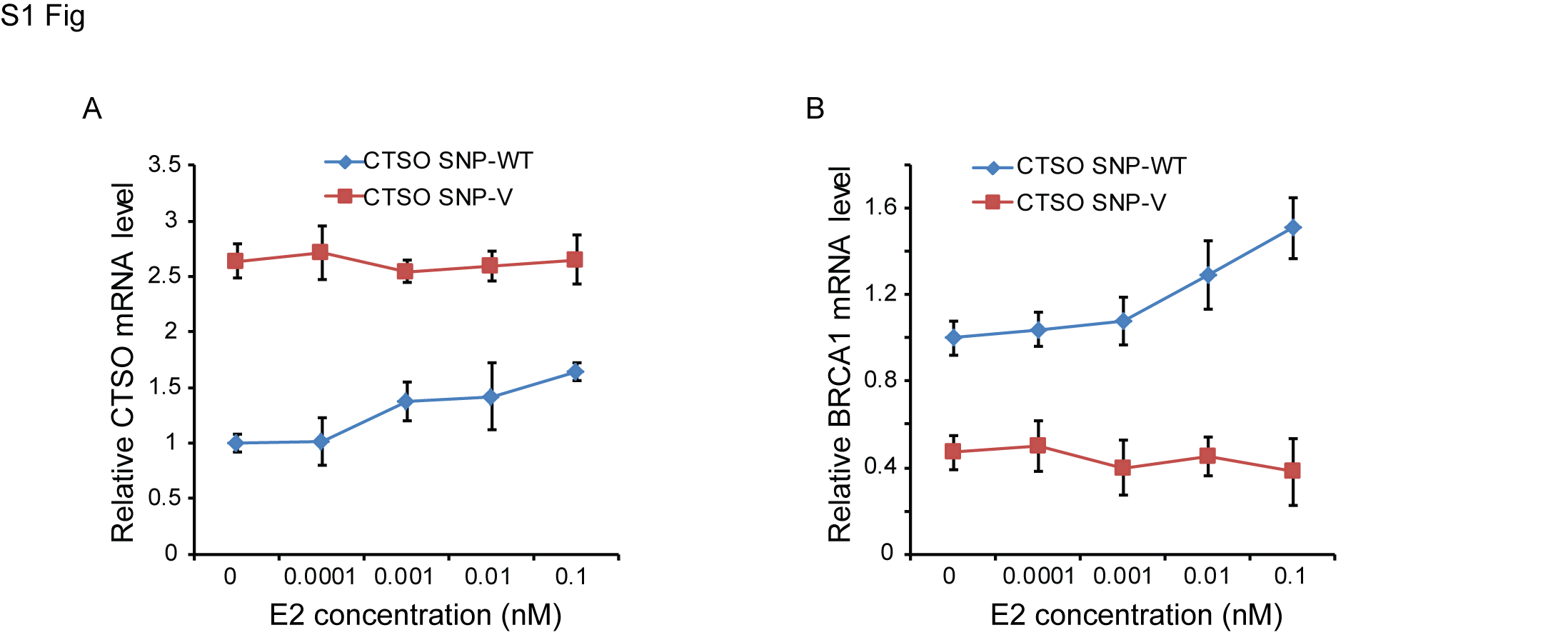

Supplement: S1 Fig — Error bars represent SEM. (TIF) [file pgen.1007031.s005.tif]

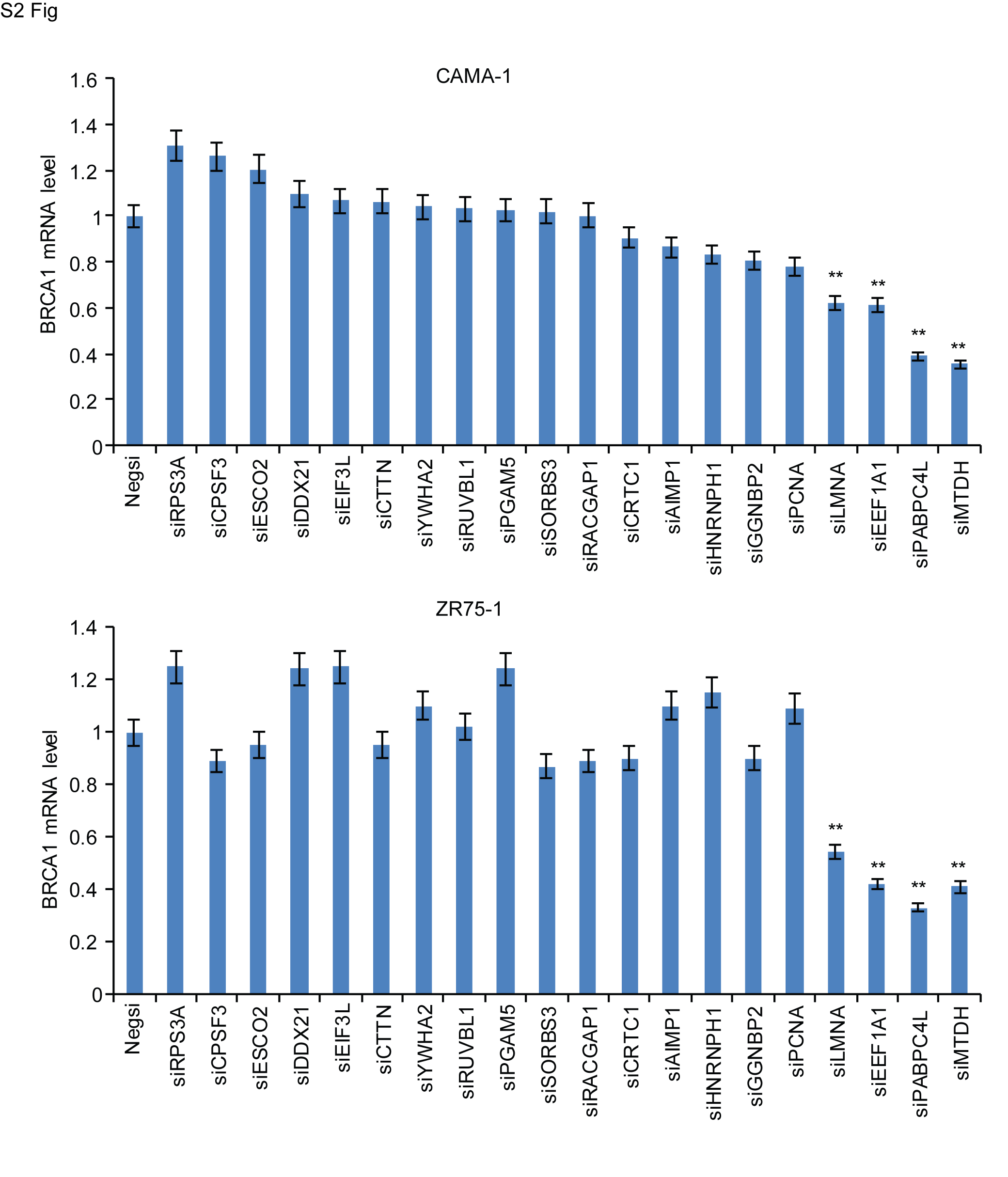

Supplement: S2 Fig — (TIF) [file pgen.1007031.s006.tif]

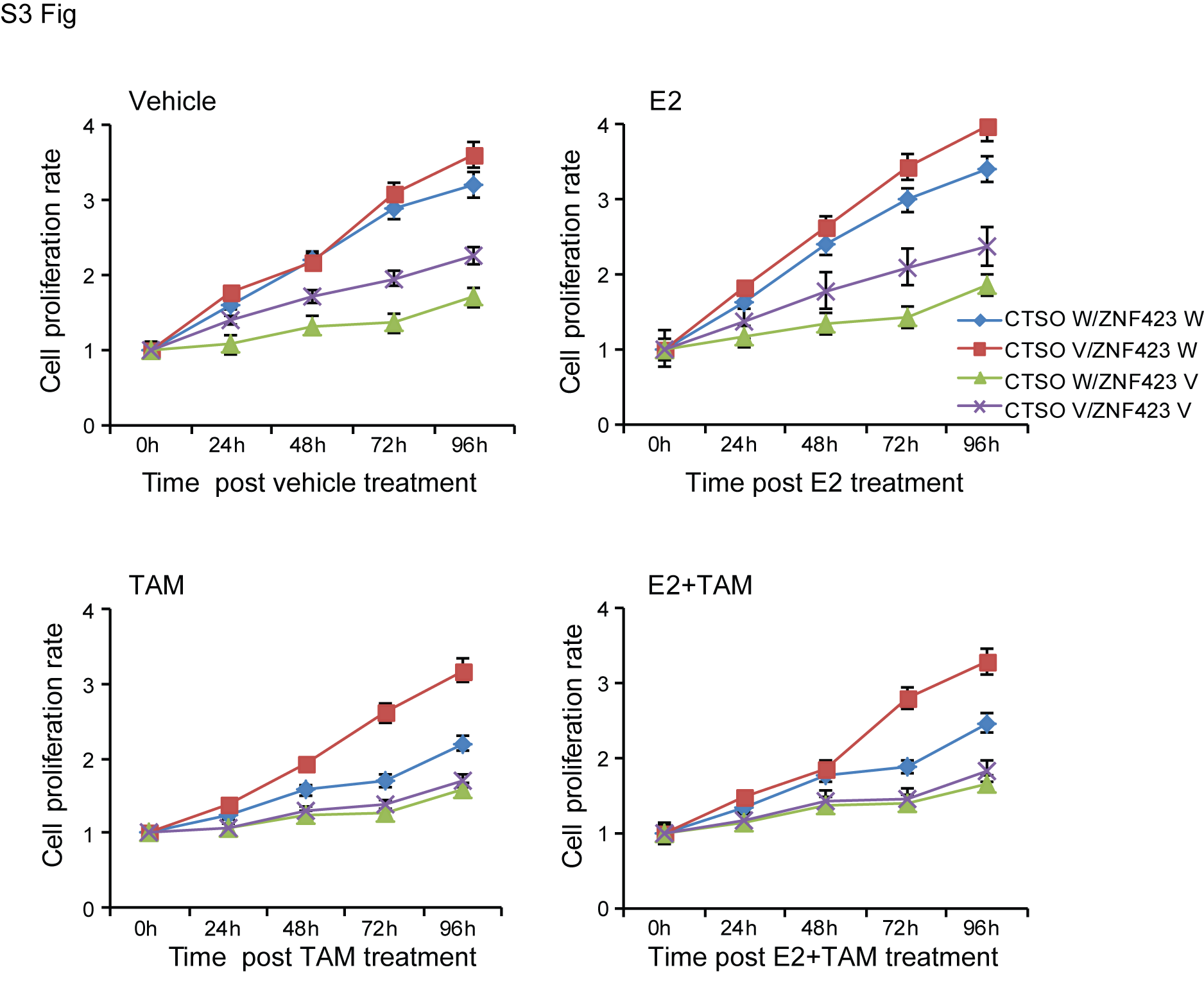

Supplement: S3 Fig — (TIF) [file pgen.1007031.s007.tif]
